# Harnessing spectra of pain psychology treatment design to improve patient access to care

**DOI:** 10.1097/PR9.0000000000001144

**Published:** 2024-04-03

**Authors:** Rachel V. Aaron, Scott G. Ravyts

**Affiliations:** Department of Physical Medicine and Rehabilitation, Johns Hopkins School of Medicine, Baltimore, MD, USA

## Abstract

**Commentary on:** Darnall BD, Burns JW, Hong J, Roy A, Slater K, Poupore-King H, Ziadni MS, You DS, Jung C, Cook KF, Lorig K, Tian L, Mackey SC. Empowered relief, cognitive behavioral therapy and health education for people with chronic pain: a comparison of outcomes at 6-month follow-up for a randomized controlled trial. PAIN Reports 2024;9:e1116.

The recent study by Darnall et al.^1^ highlights the power of diversity in pain psychology treatment design. Unlike more resource-intensive treatment options for chronic pain (eg, 8-week course of cognitive behavioral therapy [CBT] for pain), Empowered Relief represents an intervention that is more accessible and has greater potential for patient reach. The success of a single-session intervention for improving pain-related outcomes, contrasted with a traditional 8-week course of CBT, illustrates how treatments positioned at different points along spectra of treatment design—including widespread to limited in availability, general to individualized in content, and low to high in intensity—can improve pain-related outcomes. Empowered Relief exemplifies an intervention that can be widely available (ie, potential for widespread patient access), general in content (ie, fixed content delivered in a group setting), and low in intensity (ie, single session). In expanding the landscape of effective treatment options for chronic pain, the success of interventions like Empowered Relief underscores potential for widespread dissemination and raises essential questions about optimizing psychological treatment design to meet the diverse needs of patients with chronic pain in the future.

## 1. Widespread vs limited in availability

Empowered Relief—a 2-hour, single-session group-based treatment—stands out as a more readily available option compared with a traditional multiweek course of psychotherapy. Increasing accessible treatment options for people with chronic pain is crucial in our current healthcare landscape. Although pain psychology interventions are considered frontline treatments of chronic pain, the majority of patients worldwide lack access.^3^ Mental health providers trained in treating pain are limited and geographically clustered in urban areas. The widespread adoption of telehealth has reduced some geographic barriers to care; however, an unfortunate shortage of providers persists.^2^ Receiving treatment often requires a referral from a medical provider well versed in the biopsychosocial model, and once referred, patients face placement on monthslong waiting lists.^6^ Obtaining insurance reimbursement and the burden of copays or out-of-pocket expenses pose additional barriers, particularly in the United States. Examining the cost-effectiveness of Empowered Relief will shed light on scalability. Whereas single-session pain psychology interventions are likely to incur fewer costs relative to traditional multisession interventions, the exact magnitude of this difference is unclear. An effective single-session treatment is likely to be attractive to stakeholders, including referring physicians and third-party payors. Delineating its exact cost-effectiveness can be instrumental in advocating for increased coverage by insurance providers.

Empowered Relief holds promise as a way to enhance treatment access for patients and addresses several factors to further promote equitable access. In addition, as pain psychology interventions are adapted to be more readily available to patients with chronic pain, one potential challenge will be ensuring that treatment content is relevant for and inclusive of individuals from diverse cultural and socioeconomic backgrounds. Strategies such as engaging stakeholders and incorporating principles of universal design will be essential for ensuring that such interventions are not only widely available but also inclusive.^4^ As this body of work unfolds, it will be interesting to learn more about the unique facilitators and barriers to widespread and inclusive implementation and how to address them to promote equitable access to patients with chronic pain.

## 2. General to individualized in content

Empowered Relief delivers pain neuroscience education and CBT-based skills through fixed content with limited patient interaction, thereby addressing the barrier of limited trained providers and ultimately increasing its potential reach. The success of this design prompts reflection about the trade-offs of patient-to-provider interaction within traditional pain psychology treatment and patient-to-patient interaction in group-based treatments. Certainly, these are valued treatment components by many patients and providers and likely essential treatment components for many. In addition, the current findings suggest that these traditional treatment elements are not necessary to drive meaningful and lasting changes among all patients with chronic pain, raising questions about for whom which treatment elements are essential.

As this work progresses, identifying factors that moderate the success of Empowered Relief will inform the delivery of personalized pain psychology treatment. Patients facing comorbid psychosocial concerns or complex chronic pain problems may benefit from a longer course of iterative individual treatment, common to traditional courses of CBT for chronic pain. For example, in the case of comorbid chronic pain and depression, a patient may benefit from collaboration with a clinician to identify treatment targets, iterative implementation of core treatment skills, and addressing barriers to treatment adherence. Patient preference likely also plays a role. Interventions like Empowered Relief offer an interesting option for patients for whom the interactive components of traditional treatment feel too daunting and are a barrier to treatment. Stigma related to chronic pain and seeking mental health care are significant barriers to care, and the anonymity of engaging in general, vs individualized or open group, content may be preferable to some patients. Completing general treatment content may help open the door to more intensive treatment for some, helping to foster positive expectations and increase receptivity to learning. Ultimately, having interventions that span individualized to general in content holds immense value in shaping the future landscape of personalized pain psychology treatment.

## 3. Low to high intensity

The finding that a single-session dose of treatment was comparable to an 8-week dose raises questions about how to determine the ideal dose of treatment to further personalize patient care. The availability of effective single-session interventions opens the door to stepped care models, wherein all patients in a particular population (eg, patient with or at risk for developing chronic pain) receive a single-session dose of treatment and those who do not have a substantial treatment response receive a greater dose of treatment. Stepped care models maximize treatment resources, reduce patient burden of cost and time, and offer more precise care. The availability of an effective lower-intensity treatment is a critical addition to the many existing higher-intensity pain psychology treatments.

Interventions focused on pain neuroscience education are often delivered in single-session formats but often have limited efficacy for improving pain intensity and disability, particularly in the long run.^7^ By contrast, Empowered Relief was noninferior to CBT on key outcomes, including pain intensity and pain interference at 6-month follow-up. There is limited research to point to which specific components of pain psychology treatment are most effective, and the findings suggest that the authors were successful in isolating key treatment components of CBT-based skill delivery in Empowered Relief. Another interpretation of these contrasting findings is that pain neuroscience education is more likely to result in symptom reduction when combined with skill delivery.

Future research should explore the accessibility of single-session interventions for patients with lower socioeconomic resources and low health literacy. Beverly Thorn's broad body of work has identified and implemented strategies to enhance the delivery of a traditional multiweek course of CBT for chronic pain to people from low-literacy rural communities, with adaptations including presenting a limited number of concepts per session, reiterating treatment content over time, and ensuring patient comprehension.^5^ These strategies are not as readily implemented in a single-session intervention. It will be interesting to learn how single-session interventions can be adapted to ensure that the treatment content is accessible to people with low health literacy and from diverse educational backgrounds.

## 4. Conclusions

In conclusion, this study offers hope in the midst of a global chronic pain epidemic within a healthcare landscape that presents numerous barriers to receiving psychological treatment for chronic pain. It also signals a paradigm shift in pain psychology treatment, wherein investigators are meeting a need for more diversity in treatment design to facilitate widespread access to these needed treatments. Empowered Relief—which has the potential for widespread availability, is general in format, and low in intensity—offers numerous benefits to patients and has distinct implementation advantages. Looking forward, several pivotal questions emerge to ensure that interventions that are widely available, general, and low intensity meet diverse patient needs. Key themes include enhancing inclusivity and accessibility, identifying treatment moderators and ideal dosing, and assessing real-world effectiveness. The availability of an effective single-session pain psychology intervention is a valuable advancement in the field that improves accessibility of care to patients with chronic pain and offers a promising path forward in redefining how we design, deliver, and personalize psychological treatment for chronic pain.

## Disclosures

The authors have no conflict of interest to declare.
